# Coronary Subclavian Steal Syndrome With Neurological Symptoms After Coronary Artery Bypass Grafting

**DOI:** 10.7759/cureus.12833

**Published:** 2021-01-21

**Authors:** Megan C Smith, Rich Pham, Nicholas Coffey, Mohammed Kazimuddin, Aniruddha Singh

**Affiliations:** 1 Cardiology, The Medical Center/University of Kentucky, Bowling Green, USA; 2 Cardiology, University of Kentucky College of Medicine, Bowling Green, USA

**Keywords:** coronary subclavian steal syndrome, vertebral steal syndrome, coronary artery bypass graft

## Abstract

Coronary subclavian steal syndrome (CSSS) is a complication of coronary artery bypass graft surgery with the left internal mammary artery that results from left subclavian artery stenosis. A reversal of flow in the left internal mammary artery results in ischemia of the heart. We present the case of a 54-year-old man with CSSS with the rare symptom of dizziness. This indicates a potential component of undiagnosed vertebral steal syndrome as well.

## Introduction

Coronary subclavian steal syndrome (CSSS) is a complication that occurs after coronary artery bypass graft (CABG) surgery with the left internal mammary artery (LIMA). This complication arises from a reversal of flow in the grafted LIMA as a result of proximal subclavian artery stenosis or occlusion that “steals” blood from the heart. This pathological mechanism generally results in angina and upper extremity claudication [1,2]. Even with this complication, grafting the LIMA to the left anterior descending artery has become the most common graft in CABG. This is because it has superior survivability and durability compared to a saphenous vein graft (SVG).

## Case presentation

A 54-year-old Caucasian male presented to the emergency department with severe chest pain, dyspnea, and dizziness along with left arm pain and weakness. The symptoms occurred while he attempted to install an overhead light fixture. His perceived dizziness was further described as lightheadedness and presyncopal symptoms, which always occurred when he was working with his left arm, particularly if his left arm was raised above his head. He stated that his left arm pain and weakness had been progressive to the point that he could no longer work as he could not hold his tools. Previously, he had been seen in the outpatient cardiology clinic multiple times throughout the last two years with complaints of recurrent angina. However, despite medical recommendations, he never underwent a coronary angiogram or stress test for further evaluation due to severe fear and anxiety associated with these procedures.

**Figure 1:**
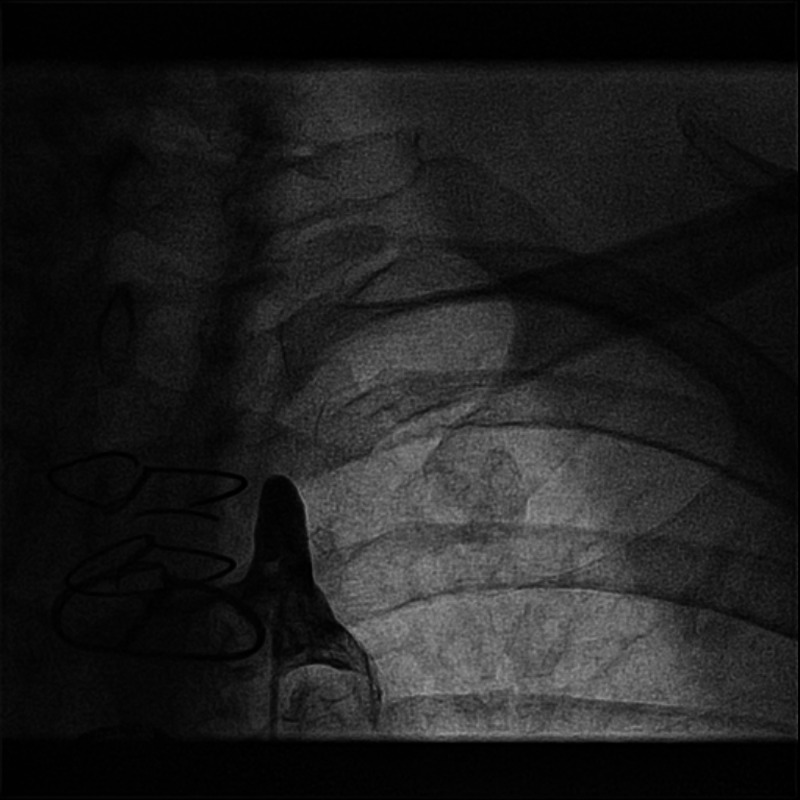
Angiogram of the proximal left subclavian artery occlusion.

Past medical history was significant for hypertension, diabetes, hyperlipidemia, smoking, anxiety, and ischemic cardiomyopathy. A component of previous treatment for the ischemic cardiomyopathy was a four-vessel CABG in 2008, achieved by grafting the LIMA to the left anterior descending artery and SVGs to a diagonal branch, obtuse marginal branch, and posterior descending artery. Vitals were significant for a 23 mmHg difference in systolic blood pressures obtained from bilateral upper extremities, and physical examination revealed diminished pulses in the left arm, raising the suspicion for subclavian stenosis. Coronary and bypass angiography was performed due to concerns of unstable angina and revealed 100% occlusion of the proximal left subclavian artery (Figure 1).

Angiogram confirmed complete flow reversal from the proximal left subclavian artery to the LIMA (Video 1).

**Video 1:** Angiogram demonstrating complete flow reversal from the proximal left subclavian artery to the LIMA. LIMA: left internal mammary artery

The LIMA had dilated from approximately its average size of 2 mm to 10 mm, which is approximately the average size of the subclavian artery. Bilateral carotid duplex revealed no hemodynamically significant stenosis. Left carotid-subclavian bypass surgery was performed which relieved his exertional angina along with dizziness and left arm discomfort.

## Discussion

CSSS was first described in 1974 and is a relatively rare disease with a prevalence of 2.5%, as defined by at least 50% left subclavian stenosis and a greater than 20 mmHg brachial pressure difference in those that have undergone LIMA CABG [3,4]. However, all patients who presented in the study with CSSS were asymptomatic [4]. Our patient presented with classic symptoms of CSSS with unstable angina (present in 30.1% of the cases) and upper extremity claudication (present in 43.3% of the cases). However, dizziness is a rare symptom that may indicate a component of vertebral steal syndrome that results in neurological symptoms. Vertebrobasilar insufficiency can manifest as syncope, dizziness, vision changes, or tinnitus [1]. Transient ischemic attacks and cerebrovascular events occur infrequently, with hemispheric events occurring most frequently in patients with concomitant carotid lesions [4]. Other case reports with neurologic symptoms describe patients presenting with isolated dizziness, recurrent vertigo, and hearing changes [5].

The key diagnostic testing for subclavian steal syndrome, which is the overarching disease that CSSS falls under, is a difference in brachial artery blood pressure of 20 mmHg, with 78-88% of those with subclavian steal syndrome having this presentation [6]. Those with higher levels of pressure difference generally have worse symptoms [7]. Imaging is used to make a definitive diagnosis through such modalities as ultrasound, magnetic radiographic imaging angiogram, and computed tomography angiography [8].

Intervention is usually performed because of potential ischemia. Intervention generally consists of either carotid-subclavian bypass surgery or endovascular therapy, such as stent placement [1]. Carotid-subclavian bypass has been shown to have operational success rates of 98.11% with 100% graft patency rate approximately two years later [9,10]. Similarly, endovascular therapy, such as stents, were reported to have 93% patency after three years for subclavian stenosis cases [9,11]. However, carotid-subclavian bypass surgery is still considered the gold standard as a direct comparison study found that stent-supported percutaneous transluminal angioplasty, which is a form of endovascular therapy, failed in 48% of occlusions, while all carotid-subclavian bypass surgeries succeeded in approximately a 50-month follow-up on average [9,12].

## Conclusions

Symptomatic CSSS is a rare complication associated with LIMA CABG that results in the “stealing” of blood by the subclavian artery. This usually presents with upper arm claudication and unstable angina as described in this case. However, this case describes an even rarer entity where CSSS was associated with neurological symptoms that could be a result of vertebral steal syndrome, which has only been reported in a few case reports in the literature.
